# Papered Chambers

**Published:** 1862-06

**Authors:** 


### PAPERED CHAMBERS.

This subject has been already referred to, but its importance may be more clearly seen and felt by considering the effects which are observed in the unfortunate poor whose lot in life is to work in coloring green leaves and buds in artificial flowers, with the same substance used in green wall-paper, the arsenite of copper, both copper and arsenic being deadly poisons. These workers are mostly women and girls, and from breathing all day the dust of this arsenite of copper, diffused throughout the atmosphere of the room, soon fall into a most deplorable condition. The derangement of the general health is all-pervading; debility, nervousness, sickness at stomach, want of appetite, thirst, headache, and looseness of bowels, the throat and gums become sore, the eyes red and weak, running of the nose, which soon becomes sore, and ugly ulcers form on the hands, face, neck, and other portions of the body; in fact, the French government, paternal in its nature, becoming acquainted with the facts, forbade the use of such materials, with the result of more beautiful colors from not unhealthful substances. An inquest was lately held on the dead body of a good-looking girl of eighteen, who had worked in these flowers; the lungs, the liver, all the tissues of the body were impregnated with arsenic. She died in great agony; the wearing of muslin masks over the nose and mouth was not sufficient to protect the lungs from the insinuating poison.

So vigilant is the French government in guarding the health and lives of the industrious poor, that a manufacturer who surreptitiously employed the poisonous and forbidden materials was, in February, 1861, fined and imprisoned, although only a slight eruption had been caused on the hands of a part of his employees. This same poisonous dust and effluvia are constantly escaping from the green paper on the walls of chambers. If there is a poisonous agent in any green color, a drop or two of elixir vitriol discolors it.

In November, 1861, a boy in his fourth year was found in convulsions, which left him apparently half-dead. Just before, the child had complained of being chilly, that he felt sick, would not take any breakfast; during the night he became exceedingly restless. His little sister was also seized with convulsions, followed by violent screaming and copious discharge of the bowels. The boy soon fell into a collapsed state, and died thirty-eight hours after the commencement of the attack. Arsenic was found in the stomach, liver, and intestines. On inquiry, it was ascertained that the children had been playing several days in a small room covered with green flock-paper; the flock brushed off readily. The quantity of the poisonous pigment was equal to one third of the weight of the paper. The coroner and his son experienced headache from sitting in a room hung with such paper. The verdict of the jury was, that the child had been poisoned by the inhalation of arsenical fumes from green paper, and that the manufacturer was guilty of very careless and culpable conduct.

There is ground for the statement that the death of Prince Albert was the result of an illness caused by occupying apartments, the atmosphere of which was contaminated by foul exhalations. Three years before, fevers of a typhoid type were prevalent at Windsor, and even in the royal apartments at the Castle. A thorough investigation was made by competent persons as to the drainage of the locality. This drainage was found most defective at those parts of the Castle where the fever appeared. This defective drainage was at the two extremities of the pile of buildings, the cloisters and the stables, for they were connected with the town sewers; the middle portion of the building had a drainage of its own, in good condition. The royal family occupied this middle portion and escaped the fever; and it is quite probable that the Prince, who was an accomplished horseman and a great lover of horses, would naturally be drawn to the stables, and thus drew "into his nostrils the breath" of death! the more easily as his constitution was not of that vigorous nature calculated to repel diseased influences. A single hour's breathing of an atmosphere loaded with miasmatic exhalations may produce deadly effects, as will appear from the following incident in another royal family, but a short time before the death of the husband of Victoria the First. It is stated in a letter from Lisbon, in reference to the death of the young King of Portugal:

"It seems the terrible malady to which the unfortunate monarch, and two of his brothers, fell victims, was caught during a visit to Alemtojo, where the air is impregnated with some miasma exceedingly dangerous to strangers. On the evening of his arrival there, the landlord of the house where the royal party had put up, came in to inquire at what hour his majesty wanted breakfast next morning, adding that it could not well be before eight, as it was very unsafe for persons not used to the air of that country to go out early, at least before sunrise; even the inhabitants never venturing abroad until the sun had dispelled the putrid vapors that arise during the night from the soil. Unmindful of this warning, the King was at his window at six next morning, asking for breakfast."

